# The Maltreatment–Aggression Link among Prosecuted Males: What about Psychopathy?

**DOI:** 10.3390/ijerph19159584

**Published:** 2022-08-04

**Authors:** Laura Woehrle, Petra Retz-Junginger, Wolfgang Retz, Steffen Barra

**Affiliations:** 1 Institute for Forensic Psychology and Psychiatry, Saarland University Hospital, Kirrberger Str., 100, 66421 Homburg, Germany; 2 Department of Psychiatry and Psychotherapy, University Medical Center, Johannes Gutenberg-University, 55131 Mainz, Germany

**Keywords:** violence, sexual offending, adverse childhood experiences, personality, trauma, offender subtypes, structural equation modeling

## Abstract

Criminal offenders constitute a high-risk sample regarding experiences of childhood maltreatment and engagement in severe aggression. Moreover, psychopathic traits are more common in samples of offenders than non-offenders. Although research has underlined the relationship between childhood maltreatment and adult aggression, the influence of psychopathy on this link is still unclear. We examined the dynamics of maltreatment, aggression, and psychopathy in a mixed sample of 239 male violent, sexual, and other offenders using latent factor structural equation modeling. We found a consistent positive association of maltreatment with aggression. Psychopathy did not mediate this relation. Maltreatment was not associated with psychopathy, although psychopathy had a positive effect on aggressive behavior. These dynamics appeared similar for violent, sexual, and other offenders. However, latent variables were constructed somewhat differently depending on the offender status. For instance, sexual abuse appeared to be of specific importance in sexual offenders. Violent offenders showed high rates of psychopathy compared to sexual and other offenders. The current findings may inspire future research to focus more closely on the different subtypes of psychopathy when examining its role in the prediction of aggression based on childhood maltreatment. Moreover, childhood maltreatment must not be neglected in treatment and prevention approaches aimed at reducing the risk of aggressive behavior.

## 1. Introduction

Childhood maltreatment, e.g., emotional, physical, and sexual abuse as well as emotional and physical neglect, can have long-lasting detrimental effects into adulthood (e.g., [1,2,3]). On a behavioral level, an increased risk of aggressive and delinquent behavior has been associated with experiences of childhood maltreatment (e.g., [4,5]). Previous studies have emphasized that childhood maltreatment not only increased the risk of aggressive and violent behavior in childhood and adolescence, but also in adulthood in terms of a dose–response relationship [6,7,8,9,10]. Offender populations constitute a particularly high-risk sample for the experience of childhood maltreatment, as well as aggressive behavior. Maltreatment prevalence rates of up to 90% have been found among offenders, with every second offender reporting more than three different kinds of maltreatment (e.g., [11]). Moreover, violent offenders reported higher levels of childhood maltreatment than non-violent offenders [12]. The type of maltreatment also appeared to be important, as, for instance, individuals with physically abusive experiences showed a higher risk of later violent behavior than individuals who had experienced sexual victimization [13]. Several theoretical frameworks have been proposed to explain the maltreatment–aggression link. According to Bandura’s social learning theory [14,15], individuals may apply aggressive behavior because they have witnessed their caregivers or other role-models successfully engaging in violence to solve interpersonal conflicts [16]. The risk of future violence may be increased even when caregivers or others do not receive any sanction for their behavior [17]. General strain theory proposes that aggression or delinquency may represent dysfunctional coping mechanisms for navigating negative emotions related to adversity and stress that are not avoidable. Early adversity, such as child maltreatment, may even lead to an enhanced vulnerability to stress, thus, increasing the risk of later aggression and violence, especially when protective coping strategies are missing [18]. Social information processing theory (SIP, [19]) suggests that aggression may result from the maladaptive processing of social cues, as maltreatment survivors may focus on hostile cues and tend towards hostile attributions of neutral cues, facilitating the retrieval of aggressive reactions thought to be effective for solving social conflicts [20]. Other researches have also stressed the role of genetic influences, as well as gene-environment interactions, in explaining the maltreatment–aggression link (e.g., [21,22]).

In addition, childhood maltreatment has been associated with psychopathic traits in adolescence and adulthood, especially in forensic populations (e.g., [4,23,24,25]). A small proportion of offenders who were high on the scale of psychopathic traits committed the majority of crimes [4,26,27]. Furthermore, the role of psychopathy appeared to be of particular importance in violent offenders [24]. It has been proposed that physical punishment and emotional neglect were related to repeated criminal conduct, antisocial behavior, and psychopathic traits [23,26]. Other studies found physical and emotional abuse, as well sexual victimization, to be strong predictors of antisocial behavior and low empathy, which both were associated with psychopathy [9,27]. Whereas some researchers stated that maltreatment influenced the behavioral rather than the affective component of psychopathy [28], others claimed that maltreatment also led to profound dysfunctional personality disturbances, culminating in antisocial personality disorder [29]. Since antisocial personality disorder and psychopathy are highly correlated, there is a reasonable probability that maltreatment may not only be associated with the behavioral outcomes of psychopathic traits, such as antisocial behavior, but also with the development of psychopathy [30,31]. In addition, neurological adaptions due to cumulative childhood adversities appeared to have negative effects on important emotional and behavioral self-regulation capacities [32]. Yet, whereas such neurological adaptions did not predict psychopathy, childhood maltreatment did [33].

In sum, the examinations of the maltreatment–aggression link, as well as the associations between maltreatment and psychopathy, on the one hand, and psychopathy and aggression, on the other hand, have been subjects of various previous studies that have given rise to ample debate that is yet to be clarified. Some researchers stated that both maltreatment and psychopathic traits were independently related to particular behavioral disturbances, such as violent and aggressive conduct (e.g., [4,27]). Others have proposed that psychopathic traits mediated the maltreatment–aggression link [31,34]. Thus, there is still a lack of knowledge regarding the dynamics of childhood maltreatment, aggression, and psychopathy, especially in offender populations, although the examination of these factors appears to be of specific importance, considering the high rates of childhood maltreatment, aggression, and psychopathic traits among criminal individuals [11,35,36,37,38,39]. Furthermore, only little is known thus far about how these dynamics may differ among different offender subtypes (e.g., violent, sexual, and other offenders).

Thus, the goal of the present study was to examine the link between childhood maltreatment and adult aggression in relation to psychopathy in a heterogeneous offender sample, focusing on the role of psychopathy as a potential mediator of the maltreatment–aggression link. Sophisticated knowledge about these dynamics may allow us to create promising interventional and preventive approaches aimed at reducing the risk of aggressive and violent behavior and, thus, contribute to the safety of our society.

Based on previous research, we expected to discover a positive predictive effect of maltreatment on aggression. Due to the lack of clarity on the role of psychopathy thus far, we examined the associations of psychopathy with both maltreatment and aggression on a rather exploratory basis. Although we did not suppose that the dynamics of maltreatment, aggression, and psychopathy would differ between offender subtypes, it seemed probable that certain adverse experiences would relate differently to the construct of maltreatment depending on the offender subtype.

## 2. Materials and Methods

### 2.1. Study Design and Procedure

The present study was conducted in the framework of an ongoing research project at the Institute for Forensic Psychology and Psychiatry, Homburg, Germany. The project aims at examining the link between childhood adversity and aggressive behavior in male offenders from different perspectives. We retrospectively analyzed data from a consecutive sample of criminal offenders who had been seen at the institute for psychological/psychiatric evaluations between August 2007 and February 2020. Evaluations were based on both (criminal and psychiatric) file contents and clinician-administered interviews, considering biographical information (including family dynamics and education), physical, mental, and sexual development, history of health problems (including substance use), as well as former and current delinquency. Moreover, offenders were asked to complete several self-rating instruments (including those used in the present study). Interviews and final evaluation reports were conducted by forensically trained and experienced psychologists and psychiatrists.

With regard to the abovementioned aims of the project, only the evaluations of those offenders who had (1) completed self-reports using the Childhood Trauma Questionnaire (CTQ, [40,41]) and the Aggression Questionnaire (AG-G, [42]), and (2) who had AQ-G inconsistency scores below 5 (see below) were considered. Starting from May 2020, the psychological/psychiatric evaluation reports of these offenders were analyzed using a specifically developed coding system based on a similar instrument that had already been successfully implemented in previous research in the context of forensic file analyses (e.g., [43,44,45]). The 44-page coding system consisted of a large number of forensically relevant variables from the following domains: (1) administrative data, (2) demographic information, (3) current/index delinquency, (4) offense analysis, (5) previous delinquency, (6) biographic/family information, (7) general and sexual development, (8) adverse childhood experiences, (9) the content of forensic evaluation, and (10) risk assessment. Retrospective analyses were conducted by trained psychologists and psychiatrists, who had not been involved in the basic evaluation process. Interrater agreement was analyzed using 30 randomly selected cases (stratified for the reason of evaluation according to criminal responsibility vs. risk assessment) that were independently double rated. The study procedures were approved by the ethics committee of the medical chamber of Saarland, Germany (No. 179/21).

### 2.2. Participants

At the time of the present study, the psychological/psychiatric evaluation reports of 239 offenders had been completely coded and were thus considered for the present analyses. Offenders were 16 to 73 years old at the time of their evaluation (M = 36.12 years, SD = 11.93 years). In 135 cases (56.6%), the offenders were evaluated for criminal responsibility, whereas 100 offenders (41.8%) were examined in the context of risk assessment. In four cases, the evaluations were aimed towards other questions, such as the inability of arrest. A total of 110 offenders (46.0%) had committed violent offenses (without sexual conduct), 61 (25.5%) has committed sexual offenses, and 68 (28.5%) had committed other (nonviolent/nonsexual) offenses (e.g., theft, fraud, or drug offenses).

### 2.3. Measurements

#### 2.3.1. Maltreatment

Childhood maltreatment was reported using the German 25-item short version of the Childhood Trauma Questionnaire (e.g., [40,41]). The CTQ is a self-rating instrument for assessing childhood maltreatment experienced up to the age of 18 in terms of emotional, physical, and sexual abuse, as well as emotional and physical neglect. Each domain is represented by 5 items investigating the frequency of maltreatment experiences between the values of 1 (“never”) and 5 (“very often”). A total score, as well as the summed scores, for each domain can be built, with higher scores representing more severe maltreatment. Previous studies emphasized the good reliability and validity of this instrument (e.g., [46,47,48]). Only the domain of physical neglect showed rather unsatisfactory internal consistencies. Similarly, Cronbach’s α was excellent for the total score and all the domain scores in the present study, except for physical neglect, which, however, still showed acceptable consistency (see Table 1).

#### 2.3.2. Aggression

Aggressive behavior was assessed using the German version of the Aggression Questionnaire (AQ-G, [42]), a self-report instrument to measure dispositional tendencies toward anger and aggression. The 34 items are rated between 1 (“not at all like me”) and 5 (“completely like me”) and assigned to five subscales: physical aggression, verbal aggression, anger, indirect aggression, and hostility. Besides the total score and domain-specific subscale scores (the higher the score is, the more severe the aggressive tendencies are), an inconsistency score indicates the probability of a response bias. According to the test manual, inconsistency scores above 4 shed considerable doubt on the honesty of the answers. International studies demonstrated good psychometric properties of the given instrument [49,50,51], whereas only moderate internal consistency was identified for the verbal aggression subscale. In the present study, Cronbach’s α indicated excellent internal consistency for the total aggression score, good internal consistencies for physical aggression, indirect aggression, and hostility, and moderate internal consistencies for verbal aggression and anger (see Table 1).

#### 2.3.3. Psychopathy

Psychopathy was retrospectively assessed based on the evaluation reports using the German version of the Psychopathy Checklist-Revised (PCL-R, [52,53]). The PCL-R is a clinician-administered instrument for assessing psychopathy, as defined by Hare, based on the file and/or interview information. Each of the 20 items can be rated as 0 (“not applicable”), 1 (“maybe applicable”), or 2 (“applicable”). Items can be summed up to a total score between 0 and 40, with scores above the cut-off of 25 points indicating a particularly high probability of having a psychopathic personality. Additionally, 4 subscales, so-called facets of psychopathy, can be derived: interpersonal, affective, lifestyle, and antisocial. The former two facets build up to one superordinate factor, the latter to a second. The manual gives instructions for correcting the facet and factor scores in the case of missing items. Several international studies have demonstrated good psychometric properties of the PCL-R (e.g., [54,55]), even when ratings were only based on file information (e.g., [53]). In the present study, the interrater agreement, as well as internal consistencies, for the facets, factors, and total scores were moderate to excellent (see Table 1).

### 2.4. Statistical Analyses

Data were analyzed in IBM SPSS and AMOS version 28.0 for Windows. Internal consistency was measured by Cronbach’s α, with α ≥ 0.60 representing acceptable and α ≥ 0.80 good consistency [56]. The interrater agreement was examined by the intra-class correlation coefficient (ICC, a two-way random, single measure, absolute agreement) using the following thresholds: 0.40–0.59 = acceptable, 0.60–0.74 = good, and >0.74 = excellent [57]. Associations between maltreatment, aggression, and psychopathy were analyzed by structural equation modeling (SEM). Maltreatment, aggression, and psychopathy were introduced as the latent variables. Maltreatment was based on the five CTQ subscale scores (emotional, physical, and sexual abuse, as well as emotional and physical neglect), aggression was based on the five AQ-G subscale scores (verbal, physical, and indirect aggression, as well as hostility and anger), and psychopathy was based on the four PCL-R facet scores (interpersonal, affective, lifestyle, and antisocial). Age at the time of the evaluation was included as a control variable in the model [58]. Missing values (due to insufficient information for the PCL-R ratings) were considered missing at random, and the data imputation was conducted using full information maximum likelihood (FIML) calculations. The model fit was estimated by several fit indices. A χ^2^/df-index < 2 indicated a good model fit [59]. Due to its sensitivity to sample size [60], we used further fit indices in addition to the χ^2^ statistics: the root mean square error of approximation (RMSEA, [61]), the comparative fit index (CFI, [62]), and the standardized root mean square residual (SRMR, [63]). According to Hu & Bentler [63], a good model fit can be assumed with RMSEA ≤ 0.06, SRMR ≤ 0.08, and CFI ≥ 0.95. CFI ≥ 0.90 was considered acceptable. We allowed indicator errors to covariate within a latent variable [64], and included covariances according to the modification indices provided by AMOS, when theoretically reasonable. For the model comparisons between the offender subtypes (i.e., violent, sexual, and other offenders), we tested for configural, metric, and scalar model invariance using the following difference thresholds: ΔCFI < 0.01, ΔRMSEA < 0.015, and ΔSRMR < 0.03 (configural to metric) or ΔSRMR < 0.01 (metric to scalar) [65,66]. Additionally, we conducted χ^2^-tests to compare the restricted and unrestricted models, as well as T-tests to compare path loadings using the Stats Tool Package [67]. We further analyzed the group differences in the manifest and latent constructs by MANOVAs with Bonferroni/Games–Howell-corrected post hoc tests. Findings of *p* < 0.05 were considered statistically significant.

## 3. Results

### 3.1. Descriptives

Table 1 displays the distribution of the abovementioned variables of interest in the total sample and in the separate violent, sexual, and other offender subtypes. Significant differences between offender subtypes on the CTQ were identified for sexual abuse (F(2, 235) = 4.51, *p* = 0.012). Post hoc tests showed that the sexual offenders had significantly higher sexual abuse scores than other offenders (M_Diff_ = 1.47, *p* = 0.004). Moreover, group differences based on the AQ-G emerged for both physical (F(2, 236) = 3.22, *p* = 0.042) and verbal aggression (F(2, 236) = 5.25, *p* = 0.006). In both cases, violent offenders showed significantly higher scores than other offenders (M_Diff_ = 2.17, *p* = 0.041 and M_Diff_ = 1.45, *p* = 0.005, respectively). Regarding psychopathy, the groups differed on the facets of affective (F(2, 229) = 4.53, *p* = 0.012), lifestyle (F(2, 229) = 6.55, *p* = 0.002), and antisocial (F(2, 229) = 8.32, *p* < 0.001), as well as factor 2 (F(2, 229) = 0.09, *p* < 0.001) and the PCL-R total score (F (2, 229) = 3.71, *p* = 0.026). Post hoc analyses revealed that with respect to the affective facet, violent and sexual offenders did not significantly differ from one another, but both differed from other offenders (M_Diff_ = 0.94, *p* = 0.016 and M_Diff_ = 0.43, *p* = 0.028, respectively), whereas, with respect to the lifestyle facet, violent offenders had significantly higher scores than sexual (M_Diff_ = 1.51, *p* = 0.002) but not other offenders. With respect to the antisocial facet, as well as factor 2, violent offenders had higher scores than sexual (M_Diff_ = 1.62, *p* < 0.001 and M_Diff_ = 3.11, *p* < 0.001, respectively) and other offenders (M_Diff_ = 1.26, *p* = 0.011, and M_Diff_ = 2.11, *p* = 0.013, respectively), who did not differ. Ultimately, violent offenders had higher PCL-R total scores than other offenders (M_Diff_ = 3.09, *p* = 0.027), which were, however, not significantly higher than the sexual offenders’ scores.

### 3.2. SEM on the Associations between Maltreatment, Aggression, and Psychopathy

We first modeled the effect of maltreatment on aggression under the control of age. The initial model showed a rather unsatisfactory fit (χ^2^ =104.66, df = 42, χ^2^/df = 2.49, CFI = 0.953, RMSEA = 0.079, SRMR = 0.044). The stepwise inclusion of error covariances between (1) emotional and physical neglect, (2) physical and sexual abuse, and (3) physical aggression and hostility resulted in a good model fit (χ^2^ = 51.37, df = 39, χ^2^/df = 1.37, CFI = 0.991, RMSEA = 0.037, SRMR = 0.037). According to this model, there was a significant positive (total) effect of maltreatment on aggression (B = 0.36, SE = 0.07, β = 0.32, *p* < 0.001). Age had a small but significant effect on aggression as well (B = −0.106, SE = 0.030, β = −0.229, *p* < 0.001).

**Figure 1:**
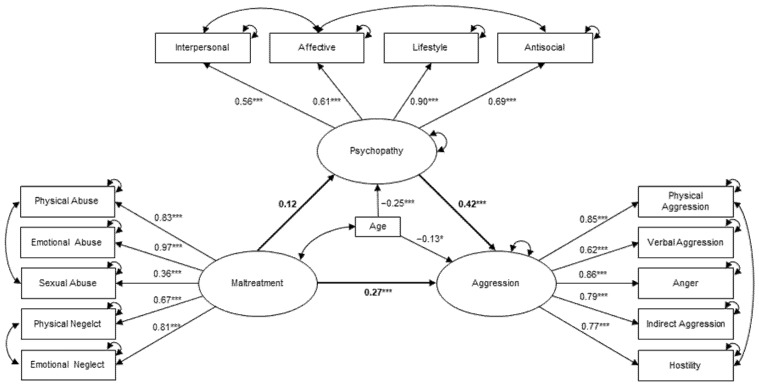
Final SEM on the associations between maltreatment, aggression, and psychopathy under the control of age. One-directional arrows show regression paths, bi-directional arrows show covariances. Standardized effects for the total sample are indicated with *** *p* < 0.001 and * *p* < 0.05.

We then added psychopathy into the model. The initial model fit was unsatisfactory (χ^2^ = 191.59, df = 82, χ^2^/df = 2.34, CFI = 0.938, RMSEA = 0.075, SRMR = 0.071). The stepwise inclusion of error covariances between the facets of interpersonal and affective, as well as affective and antisocial, resulted in a good model fit (χ^2^ = 153.23, df = 80, χ^2^/df = 1.92, CFI = 0.959, RMSEA = 0.062, SRMR = 0.067). The final model is shown in Figure 1.

The direct effect of maltreatment on aggression remained significant but was slightly smaller than the total effect (B = 0.30, SE = 0.07, β = 0.27, *p* = 0.002). However, the path loading between maltreatment and psychopathy did not show significance (B = 0.03, SE = 0.01, β = 0.12, *p* = 0.083). On the other hand, psychopathy was significantly associated with aggression (B = 2.28, SE = 0.44, β = 0.42, *p* = 0.002). No significant indirect (mediated) effect of maltreatment on aggression was identified (B = 0.06, SE = 0.04, β = 0.05, *p* = 0.071). Age had small but significant influences on both aggression (B = −0.06, SE = 0.03, β = −0.13, *p* < 0.036) and psychopathy (B = −0.02, SE = 0.01, β = −0.25, *p* < 0.001).

### 3.3. Differences between Offender Subtypes

The abovementioned SEM showed an acceptable model fit when the offender subtypes (violent, sexual, and other offenders) were considered (χ^2^ = 358.20, df = 240, χ^2^/df = 1.49, CFI = 0.935, RMSEA = 0.046, SRMR = 0.081, indicating configural invariance). Full metric invariance was not established (χ^2^ = 420.058, df = 262, χ^2^/df = 1.6032, CFI = 0.913, RMSEA = 0.051, SRMR = 0.093). Instead, the model showed a rather partial metric invariance when the path between sexual abuse and maltreatment remained unrestricted (χ^2^ = 404.915, df = 260, χ^2^/df = 1.575, CFI = 0.920, RMSEA = 0.049, SRMR = 0.090). Equally, we did not find full scalar (χ^2^ = 475.084, df = 286, χ^2^/df = 1.661, CFI = 0.896, RMSEA = 0.053, SRMR = 0.088) but, rather, partial scalar invariance when the intercepts for the PCL-R affective facet and the AQ-G verbal aggression subscale remained unrestricted (χ^2^ = 441.078, df = 282, χ^2^/df = 1.564, CFI = 0.912, RMSEA = 0.049, SRMR = 0.088).

Differences in the particular paths of the model are shown in Table 2. A χ^2^-difference test between the final model and the model with restrictions on the sexual abuse-maltreatment path across offender subtypes showed significant differences (Δχ^2^(2) = 15.323, *p* < 0.001). The effects of maltreatment on sexual abuse were significant in each of the three offender groups, but larger in the sexual offender subtype than in violent or other offenders, whereas the latter groups did not differ significantly.

Similar to the results regarding the total sample, the significant direct effect of maltreatment on aggression was identified in each offender subtype. No significant indirect effects of maltreatment on aggression were identified in any offender subtype (all were *p* > 0.05). When the path between maltreatment and aggression was set as equal across groups, the model fit worsened only slightly (χ^2^ = 442.913, df = 284, χ^2^/df = 1.56, CFI = 0.913, RMSEA = 0.049, SRMR = 0.088). No differences emerged from the χ^2^-difference test (Δχ^2^(2) = 1.835, *p* = 0.400) and, thus, in the path coefficients across groups. Moreover, there was no significant relation between maltreatment and psychopathy in any offender subtype. When the path between maltreatment and psychopathy was set as equal across groups, the model fit worsened as well (χ^2^ = 441.490, df = 284, χ^2^/df = 1.56, CFI = 0.913, RMSEA = 0.048, SRMR = 0.088). Again, no differences emerged from the χ^2^-difference test (Δχ^2^(2) = 0.412, *p* = 0.521) and between the path loadings across groups. Ultimately, the link between psychopathy and aggression appeared significant in each of the offender subtypes. When the path between psychopathy and aggression was set as equal across groups, the model fit slightly worsened (χ^2^ = 443.744, df = 284, χ^2^/df = 1.56, CFI = 0.912, RMSEA = 0.049, SRMR = 0.090). Once again, no significant differences emerged from the χ^2^-difference test (Δχ^2^(2) = 2.67, *p* = 0.264) and between the path loadings across groups.

Finally, we examined whether the mean latent variable scores differed significantly between the offender groups. No differences were found for the maltreatment (F(2, 236) = 0.62, *p* = 0.541) or aggression scores (F(2, 236) = 1.45, *p* = 0.238), but they were for psychopathy (F(2, 238) = 5.94, *p* = 0.003). Post hoc analyses revealed that latent psychopathy was considerably higher in violent compared to sexual offenders (M_Diff_ = 0.51, *p* = 0.005). However, slight differences emerged between violent and other offenders (M_Diff_ = 0.37, *p* = 0.050), and no differences were found between sexual and other offenders (M_Diff_ = −0.13, *p* = 1.00).

## 4. Discussion

### 4.1. General Findings

The present study examined the associations between the forensically relevant constructs of childhood maltreatment, aggression, and psychopathy among males prosecuted for violent, sexual, and other offenses. As expected, childhood maltreatment positively predicted an adult tendency toward aggression. Although aggression appeared to decline with increasing age, the effect of maltreatment on aggression remained significant, even under the statistical control of age. These findings confirm previous research findings, indicating that childhood maltreatment can be a critical risk factor for aggressive and delinquent behavior (e.g., [4]) and that the probability of aggression increases with elevating rates of adverse childhood experiences (e.g., [7,8]). Concerning the age effects, prior studies have shown that childhood maltreatment was consistently associated with aggression over the lifespan [9], a finding which was supported by the present results.

We did not find any associations between childhood maltreatment and psychopathy. Although psychopathy positively predicted aggression, it did not mediate the maltreatment–aggression link. Whereas the relationship between psychopathy and aggression has often been highlighted and appears understandable, considering that the definition of psychopathy includes, among other factors, antisocial behavior and the reckless satisfaction of one’s needs, the link between maltreatment and psychopathy has been subject to ample debate. Some studies indicated that maltreatment was related to the antisocial domain of psychopathy, but not to the interpersonal and affective facets [35,68]. According to the differentiation between primary and secondary psychopathy [69,70], the interpersonal and affective components may be more reflective of primary psychopathy, which seems to be innate and genetically conditioned, whereas secondary psychopathy develops through aversive experiences, such as maltreatment, by establishing emotional detachment or dullness as a response to cope with severe stress. This can, however, reflect some kind of pathological adaption that increases the risk of becoming a perpetrator of violence oneself [70]. Furthermore, secondary psychopathy has been shown to often be accompanied by increased impulsivity, hostility, and reactive aggression [70,71]. Metcalf and colleagues [72] found considerably high rates of maltreatment, as well as aggression, in youths who were high on the scale of secondary psychopathic traits compared to youths high on the scale of primary psychopathic traits. However, most studies that have found associations between maltreatment and psychopathic traits have focused on the behavioral components of psychopathy or have not differentiated sufficiently between psychopathy and antisocial behavior/personality disorder. Thus, we might have failed to detect more detailed associations between childhood maltreatment and the specific subtypes of psychopathy, since we relied on a rather comprehensive view of psychopathy as a latent construct.

### 4.2. Differences among Offender Subtypes

The abovementioned relationships between maltreatment, aggression, and psychopathy in the total sample also appeared when offender subtypes were separately examined. Thus, it can be assumed that the maltreatment–aggression link, as well as the association of psychopathy with aggression, are independent of offender status. Although the path coefficients slightly differed, no statistical significance resulted from these differences. However, when discussing the differences in abovementioned associations between violent, sexual, and other offenders, it is worth mentioning that the model used in the present study only showed partial metric and scalar invariance. Thus, the structure of the respective latent constructs appeared to differ between groups. Indeed, sexual abuse was particularly prevalent in sexual offenders. Previous studies have indicated that certain forms of maltreatment may have differential effects. For instance, sexual abuse has often been linked to later sexual offending [16,73,74,75]. Glasser et al. [76] found that, in a sample of sexual offenders, 35% had reported sexual victimization, whereas non-delinquent controls only showed a sexual abuse prevalence of 11%. However, research has also highlighted that only a minority of sexual abuse survivors also become sexual offenders [75,77]. There are some findings indicating that it is the pattern of different kinds of maltreatment, often including sexual abuse, rather than sexual abuse alone that increases the risk of sexual offending over the lifespan [43,75]. Thus, maltreatment in general appears to be a risk factor for criminal conduct [7,8,12]. The current findings confirm prior research findings, as latent maltreatment was significantly related to all the considered indicators in each offender subtype. Moreover, the latent maltreatment and latent aggression scores did not differ between offender groups, although, on the indicator level, violent offenders showed comparatively high scores for physical and verbal aggression. Once again, the partial model invariance indicates that the latent variables may be constructed differently, depending on the offender status. Moreover, violent offenders had the highest latent psychopathy scores. Also on the indicator level, violent offenders stood out for their high psychopathy scores, especially in terms of antisocial behavior. As mentioned above, psychopathy has often been related to aggression and violent offending (e.g., [4,35,36]). Not only did offenders high on the psychopathy scale exhibit more severe violent criminal careers [78], but high levels of psychopathy have also been repeatedly associated with an increased risk of violent reoffending [53,55].

### 4.3. Strenghts and Limitations

The present results must not be interpreted without considering several strengths and qualifications. Firstly, the present study was based on a heterogeneous sample of adult offenders, which allowed comparisons between violent, sexual, and other offender subtypes to be made. However, by only considering offenders referred to one forensic institute in Germany, the generalizability of our findings is limited. Furthermore, due to the study design restrictions, we were not able to consider reports on female offenders, which would have allowed us to examine potential gender effects, too. There is some empirical evidence to support the hypothesis that the link between maltreatment and delinquency may be stronger for males than females [73], and that psychopathy may be more frequent in males than females [79]. It has been assumed that some facets of psychopathy, such as antisocial behavior, may come to be expressed differently depending on gender, e.g., in terms of lower frequencies of aggressive and violent acts but higher rates of sexual permissiveness and excessive lying in females compared to males [80]. Secondly, we were able to assess both self-rated and clinician-administered information using instruments that have been repeatedly validated with good psychometric properties. All the evaluation reports were created and coded by trained and experienced psychiatric/psychological staff, which ensured high data quality. Yet, self-reports always bear the risk of response bias, e.g., due to social desirability. On the one hand, we aimed to achieve greater data validity by excluding those offenders with critical inconsistency scores on the AQ-G from the beginning. On the other hand, this decision might have introduced the risk of some selection bias. Thirdly, assessing psychopathy by the often-used PCL-R appeared to be preferable in the present study because—according to its authors—it can be coded based on file information only. However, whereas the interrater agreement was good for the lifestyle and antisocial (factor 2) facets of psychopathy, it was only acceptable for the interpersonal and affective (factor 1) facets. This might be due to the fact that the latter, especially, require information derived from direct communication and are difficult to code based on file content (evaluation reports) alone. Some previous studies have found similar tendencies (e.g., [81]). Fourthly, although the total sample size was sufficient for implementing SEM [58], the subsamples were smaller, raising concerns about their statistical power. However, most of the fit indices used in the present study have been shown to be fairly independent of the sample size (e.g., [65]). Fifthly, as mentioned above, the comparison of the latent constructs across offender subtypes is limited, because the models did not reach full metric or scalar invariance, which indicated that the latent variables might be somewhat differently constructed across offender subtypes. Yet, full invariance is rather uncommon. According to Meade et al. [82], variations of the parameters are tenable if at least two indicators are invariant. Since this was the case in the present study, the latent means and their associations may be compared according to the abovementioned qualifications. Ultimately, the inclusion of psychiatric disorders was beyond the scope of the present study, although there may have been some confounding effects of mental health problems on the examined associations. There has been some debate about the role of psychiatric disorders in the dynamics between maltreatment and aggression. Whereas some studies found mediating effects of psychopathology on the maltreatment–aggression link [83], others emphasized that certain types of maltreatment predicted later violent/delinquent behavior over and above the effects of mental health [84]. Future studies should consider these effects more carefully.

## 5. Conclusions

Our findings indicate that maltreatment may not be associated with overall psychopathy, but that a more detailed investigation is required to further explain the associations of maltreatment with both psychopathy and aggression, e.g., by differentiating between primary and secondary psychopathy. Moreover, the subtypes of aggression could be included. For instance, DeLisi et al. [35] found that the associations between maltreatment and proactive aggression were indeed mediated by psychopathic traits. Moreover, the construct of maltreatment could be broadened to include patterns of other types of adverse childhood experiences in both intra- and extra-familial contexts to explore their role in aggressive and delinquent behavior. In addition to inspiring future research, our results allow suggestions for intervention and prevention to be made. Similar to previous research (e.g., [4,85]), considerably high rates of childhood adversity were found among offenders, which were significantly associated with elevated aggressive tendencies. Thus, treatment aimed at reducing aggressive and criminal conduct must not neglect the consideration of childhood maltreatment. One treatment approach focusing on both adverse experiences and aggressive behavior is narrative exposure therapy for forensic offender rehabilitation (FORNET, [86]). FORNET has been shown to be effective among severe violent offenders in terms of increased mental health, reduced violent reoffending risk, and successful reintegration into society [87].

In conclusion, the current findings support previous research findings, emphasizing the predictive associations between childhood maltreatment and later aggressive tendencies, as well as psychopathy and aggression, in a heterogenous offender sample, although mediating effects of psychopathy on the maltreatment–aggression link were not derivable. Empirically based knowledge of these relationships and their potential consequences is important, as it enables the deduction of adequate intervention and prevention approaches. Thus, informed therapists may help clients to counteract maladaptive effects and, eventually, to reduce their risk of engaging in repeated aggressive and criminal conduct.

## Figures and Tables

**Table 1:** Distribution of Variables of Interest in the Total Sample and in Offender Subtypes.

		Total Sample (*n* = 239)				Violent Offenders (*n* = 110)				Sexual Offenders (*n* = 61)				Other Offenders (*n* = 68)			
Scale		Cronbach’s α	*M*	SD	Range	Cronbach’s α	*M*	SD	Range	Cronbach’s α	*M*	SD	Range	Cronbach’s α	*M*	SD	Range
**CTQ**	Emotional Abuse	0.94	9.34	5.20	5.00–25.00	0.95	9.45 ^a^	5.43	5.00–25.00	0.94	9.75 ^a^	5.78	5.00–25.00	0.91	8.79 ^a^	4.20	5.00–21.00
	Physical Abuse	0.94	8.16	4.93	5.00–25.00	0.95	8.62 ^a^	5.40	5.00–25.00	0.94	8.59 ^a^	5.17	5.00–23.00	0.89	7.04 ^a^	3.62	5.00–21.00
	Sexual Abuse	0.96	5.81	2.82	5.00–25.00	0.99	5.75 ^a,b^	3.21	5.00–25.00	0.92	6.62 ^a^	3.34	5.00–21.00	0.78	5.18 ^b^	0.73	5.00–9.00
	Emotional Neglect	0.96	11.57	6.03	5.00–25.00	0.97	11.77 ^a^	6.37	5.00–25.00	0.96	11.02 ^a^	6.02	5.00–23.00	0.95	11.62 ^a^	5.51	5.00–24.00
	Physical Neglect	0.74	9.69	4.05	5.00–21.00	0.83	9.71 ^a^	4.57	5.00–21.00	0.71	9.72 ^a^	3.94	5.00–21.00	0.52	9.62 ^a^	3.21	5.00–21.00
	Total Score	0.96	44.60	19.07	25.00–114.00	0.97	45.31 ^a^	20.97	25.00–114.00	0.96	45.92 ^a^	20.20	25.00–97.00	0.93	42.29 ^a^	14.29	25.00–88.00
**AQ-G**	Physical Aggression	0.88	14.68	6.56	8.00–36.00	0.88	15.83 ^a^	7.06	8.00–36.00	0.91	13.74 ^a, b^	7.08	8.00–35.00	0.78	13.66 ^b^	4.79	8.00–29.00
	Verbal Aggression	0.61	12.97	3.04	6.00–23.00	0.67	13.44 ^a^	3.19	6.00–23.00	0.44	13.21 ^a, b^	2.78	7.00–21.00	0.58	11.99 ^b^	2.80	7.00–20.00
	Anger	0.73	13.65	4.67	7.00–32.00	0.72	14.00 ^a^	4.81	7.00–29.00	0.79	13.43 ^a^	4.97	7.00–32.00	0.70	13.29 ^a^	4.16	7.00–28.00
	Indirect Aggression	0.80	11.79	4.26	6.00–28.00	0.82	11.91 ^a^	4.16	6.00–23.00	0.87	12.03 ^a^	5.10	6.00–28.00	0.62	11.37 ^a^	3.68	6.00–21.00
	Hostility	0.81	17.27	5.65	8.00–36.00	0.81	17.22 ^a^	5.42	8.00–33.00	0.83	17.18 ^a^	5.64	8.00–36.00	0.78	17.43 ^a^	6.11	8.00–36.00
	Total Score	0.94	70.36	20.03	38.00–146.00	0.94	72.40 ^a^	20.80	38.00–140.00	0.95	69.59 ^a^	21.75	48.00–146.00	0.92	67.74 ^a^	16.84	42.00–130
**PCL-R**	Interpersonal	0.76	1.39	1.84	0.00–8.00	0.82	1.40 ^a^	1.98	0.00–8.00	0.76	1.25 ^a^	1.78	0.00–8.00	0.66	1.51 ^a^	1.65	0.00–7.00
	Affective	0.85	2.76	2.30	0.00–8.00	0.84	2.97 ^a^	2.20	0.00–8.00	0.86	3.16 ^a^	2.62	0.00–8.00	0.85	2.07 ^b^	2.03	0.00–8.00
	Lifestyle	0.79	3.44	2.71	0.00–10.00	0.81	4.06 ^a^	2.85	0.00–10.00	0.80	2.61 ^b^	2.63	0.00–10.00	0.69	3.18 ^a,b^	2.33	0.00–10.00
	Antisocial	0.75	3.41	2.78	0.00–10.00	0.76	4.15 ^a^	2.88	0.00–10.00	0.74	2.60 ^b^	2.47	0.00–09.00	0.72	2.95 ^b^	2.56	0.00–8.70
	Factor 1	0.86	4.17	3.71	0.00–16.00	0.87	4.39 ^a^	3.76	0.00–16.00	0.86	4.42 ^a^	3.93	0.00–15.00	0.85	3.58 ^a^	3.43	0.00–15.00
	Factor 2	0.83	6.89	4.97	0.00–20.00	0.85	8.25 ^a^	5.16	0.00–20.00	0.82	5.20 ^b^	4.60	0.00–18.00	0.77	6.21 ^b^	4.37	0.00–18.00
	Total Score	0.87	11.74	7.91	0.00–37.00	0.88	13.17 ^a^	7.97	0.00–37.00	0.88	10.84 ^a,b^	8.15	0.00–35.00	0.84	10.16 ^b^	7.26	0.00–33.00

Note. Different superscripts indicate statistically significant differences among groups (*p* < 0.05).

**Table 2:** SEM path loadings across offender subtypes.

SEM Path	Violent Offenders (*n* = 110)			Sexual Offenders (*n* = 61)			Other Offenders (*n* = 68)		
	B	SE	β	B	SE	β	B	SE	β
Maltreatment → Sexual Abuse	0.16 **^a^	0.06	0.27	0.35 ***^b^	0.07	0.56	0.05 *^a^	0.02	0.29
Maltreatment → Aggression	0.28 **^a^	0.10	0.25	0.29 *^a^	0.15	0.25	0.490 ***^a^	0.13	0.43
Maltreatment → Psychopathy	0.01 ^a^	0.02	0.07	0.03 ^a^	0.03	0.13	0.03 ^a^	0.03	0.24
Psychopathy → Aggression	2.85 ***^a^	0.63	0.49	2.05 **^a^	0.72	0.44	1.51 *^a^	0.60	0.30

Note. * *p* ≤ 0.05, ** *p* ≤ 0.01, *** *p* ≤ 0.001. B = unstandardized regression weight, SE = standard error, β = standardized regression weight. Different superscripts indicate statistically significant differences among groups.

## Data Availability

The data presented in this study are available on request from the last author (S.B.).
